# New Appliances and Things Medical

**Published:** 1903-08-08

**Authors:** 


### NEW APPLIANCES AND THINGS MEDICAL.

[We shall be glad to receive at our Office, 28 & 29 Southampton Street, Strand, London, W.C., from the manufacturers, specimens of all new preparations and appliances which may be brought out from time to time.]

### SAVORE.

(SAVORY AND MOORE, LIMITED, 143 NEW BOND STREET, LONDON, W.)

THIS food is scientifically prepared to meet the requirements of those experiencing digestive difficulties or unable to assimilate ordinary nourishment. It contains soluble milk and cereal proteids and albumoses, together with carbohydrates, so blended as to contain all the necessary elements of nutrition in their requisite proportion. Its capability of lending itself to a variety of dishes is useful, while the name of Savory and Moore is sufficient guarantee of the purity of the ingredients.

### CHLOROS.

(THE UNITED ALKALI COMPANY, LIMITED, 30 JAMES STREET, LIVERPOOL.)

AT one time chlorine was neglected owing to the difficulty of handling it. Now, however, special study has produced Chloros, a valuable disinfectant and deodoriser. It is a lightish brown liquid, containing 10 per cent. by weight of available chlorine. A strength of 1 in 2,000 is sufficient to kill the germs of scarlet fever and enteric fever in a few minutes, while a strength of 1 in 5,000 will destroy the bacillus coli communis in 15 minutes. Its precipitation but non-coagulation of albumen is certainly much in its favour. "Chloros" is the most economical and practical medium for soaking "typhoid linen." We highly recommend its wide use both for private and public sanitary purposes.

### RENINE.

(RENINE LTD., 254A HIGH HOLBORN, LONDON, W.C.)

THIS is a medicinal water from Lichtenberg springs containing a large proportion of pot. nitrate with a low proportion of solids held in solution by carbonic acid, besides some ferruginous salts. Being free from micro-organisms and containing no objectionable organic matter, it will doubtless be useful in cases where a diuretic water is required.

